# A Randomized Controlled Phase 3 Study on the Efficacy and Safety of Recombinant Human Growth Hormone in Children With Idiopathic Short Stature

**DOI:** 10.3389/fendo.2022.864908

**Published:** 2022-04-29

**Authors:** Jinna Yuan, Junfen Fu, Haiyan Wei, Gaixiu Zhang, Yanfeng Xiao, Hongwei Du, Wei Gu, Yanhong Li, Linqi Chen, Feihong Luo, Yan Zhong, Haihong Gong

**Affiliations:** ^1^ Endocrinology Department, Children’s Hospital of Zhejiang University School of Medicine, National Clinical Research Center for Child Health, Hangzhou, China; ^2^ Department of Endocrinology, Genetics and Metabolism, Zhengzhou Children’s Hospital, Zhengzhou, China; ^3^ Department of Pediatrics and Endocrinology, Children’s Hospital of Shanxi, Taiyuan, China; ^4^ Department of Pediatrics, Second Affiliated Hospital of Xi’an Jiaotong University, Xi'an, China; ^5^ Department of Pediatrics and Endocrinology, The First Hospital of Jilin University, Jilin, China; ^6^ Department of Endocrinology, Nanjing Children’s Hospital, Nanjing, China; ^7^ Department of Pediatrics, The First Affiliated Hospital, Sun Yat-sen University, Guangzhou, China; ^8^ Department of Endocrinology, Genetics and Metabolism, Children’s Hospital of Soochow University, Suzhou, China; ^9^ Department of Endocrinology, Children’s Hospital of Fudan University, Shanghai, China; ^10^ Children Health Division, Hunan Children’s Hospital, Changsha, China; ^11^ Department of Pediatrics, Jiangsu Provincial People’s Hospital, Nanjing, China

**Keywords:** efficacy, safety, rhGH, idiopathic short stature, China

## Abstract

**Background:**

To evaluate the safety and efficacy of daily somatropin (Jintropin^®^), a recombinant human growth hormone, in prepubertal children with ISS in China.

**Methods:**

This study was a multicenter, randomized, controlled, open-label, phase 3 study. All subjects were randomized 3:1 to daily somatropin 0.05 mg/kg/day or no treatment for 52 weeks. A total of 481 subjects with a mean baseline age of 5.8 years were enrolled in the study. The primary endpoint was change in (△) height standard deviation score (HT-SDS) for chronological age (CA). Secondary endpoints included △height from baseline; △bone age (BA)/CA; △height velocity (HV) and △insulin-like growth factor 1 (IGF-1 SDS).

**Results:**

△HT-SDS at week 52 was 1.04 ± 0.31 in the treatment group and 0.20 ± 0.33 in the control group (*P* < 0.001). At week 52, statistical significance was observed in the treatment group compared with control for △height (10.19 ± 1.47 cm vs. 5.85 ± 1.80 cm; *P* < 0.001), △BA/CA (0.04 ± 0.09 vs. 0.004 ± 0.01; *P* < 0.001), △HV (5.17 ± 3.70 cm/year vs. 0.75 ± 4.34 cm/year; *P* < 0.001), and △IGF-1 SDS (2.31 ± 1.20 vs. 0.22 ± 0.98; *P* < 0.001). The frequencies of treatment-emergent adverse events (TEAEs) were similar for the treatment and the control groups (89.8% vs. 82.4%); most TEAEs were mild to moderate in severity and 23 AEs were considered study-drug related.

**Conclusions:**

Daily subcutaneous administration of somatropin at 0.05 mg/kg/day for 52 weeks demonstrated improvement in growth outcomes and was well tolerated with a favorable safety profile.

**Trial Registration:**

ClinicalTrials.gov (identifier: NCT03635580). URL: https://clinicaltrials.gov/ct2/show/NCT03635580

## 1 Introduction

Idiopathic short stature (ISS) refers to a condition characterized by a height more than 2 standard deviation score (SDS) below the corresponding mean height for a given age, gender, and population that has no evidence of underlying pathology (1). ISS accounts for 80% of children with short stature of a height below –2 SDS (1). In 2 retrospective, single-center studies, ISS was found in approximately 40% of the study population (2, 3).

The use of growth hormone (GH) was approved by the U.S. Food and Drug Administration (FDA) in 2003 for children with ISS with a height of more than 2.25 SDS below the mean height and who are unlikely to attain normal adult height (4). A consensus statement published by the Growth Hormone Research Society, the Lawson Wilkins Pediatric Endocrine Society, and the European Society for Paediatric Endocrinology recommended that children with ISS at a height of less than –2 SDS and were also more than 2 SDS below midparental height could be treated with GH (5).

The cause of ISS remains unknown and children with ISS have normal birth weight and GH levels. It is postulated that it is due to genetic aberrations along the GH-insulin-like growth factor 1 (IGF-1) pathway and in the short stature homeobox-containing (SHOX) gene (4). The purpose of treatment is to enable individuals with ISS to attain normal, or close to normal, adult height and avoid any psychological issues that come with extreme or unacceptable short stature. However, individual responses to GH are highly variable; treatment is considered successful if, in the first year, a change in (△) height SDS (HT-SDS) of more than 0.3–0.5 and an increment in height velocity (HV) of more than 3 cm/year is achieved (5).

Longer-term treatment with GH has been reported to increase mean adult height by 3.5–7.5 cm in children with ISS and had a safety profile similar to outcomes in other GH disorders; most adverse events (AEs) were mild in severity with a low risk of high-grade toxicities (5). In China, there were several studies demonstrating the clinical benefit of recombinant human GH (rhGH) therapy in children with ISS compared with baseline (6, 7). However, most of these studies were retrospective and observational by design. There is a lack of clear data on the effectiveness and safety of rhGH therapy in children with ISS in China.

Somatropin (Jintropin^®^, GeneScience Pharmaceuticals, Changchun, China) is a daily rhGH therapy that was approved by the China FDA in 2005 for the treatment of GH deficiency, severe burns, Noonan syndrome, short stature caused by SHOX deficiency, achondroplasia, gonad hypoplasia (Turner syndrome), children small for gestational age (failure to catch-up growth at age 2 years), hypothalamic-pituitary disorder caused by GH deficiency, and short bowel syndrome in patients receiving specialized nutritional support. Somatropin has demonstrated safety and efficacy in all the approved indications.

We conducted a phase 3 study to evaluate the safety and efficacy of daily somatropin in prepubertal children with ISS in China.

## 2 Methods

### 2.1 Subjects

Inclusion criteria were: 1) aged between 4–9 years in girls and 4–10 years in boys; 2) HT-SDS ≤–2.25 SD of the average height of normal children of the same age and gender based on the Chinese general population at the time of screening (8); 3) peaked stimulated GH ≥10 ng/mL; 4) bone age (BA) ≤ actual age + 6 months; 5) prepubertal (Tanner stage 1); and 6) no previous history of GH treatment.

Exclusion criteria were: 1) liver or kidney dysfunction; 2) positive for hepatitis B virus; 3) known allergy to the investigational product; 4) systemic chronic disease or immune deficient; 5) diagnosed with, or at high risk of, malignancy; 6) mental illness; 7) diagnosed with other growth and development disorders (GH deficiency, Turner syndrome, Noonan syndrome, Laron syndrome, small for gestational age, or growth disorders caused by malnutrition or hypothyroidism, or short stature of other known causes); 8) impaired glucose regulation or diabetes; 9) body mass index ≥22 kg/m^2^; 10) congenital skeletal abnormalities, scoliosis, or claudication; 11) participated in other clinical trials within 3 months; 12) received medication or other hormones that may interfere with GH secretion or function; and 13) deemed inappropriate by the study investigators. Magnetic resonance imaging (MRI) scans of the pituitary gland were conducted to exclude pituitary tumors.

### 2.2 Study Design

This phase 3 study consisted of 2 phases. The first phase was a 52-week, multicenter, randomized, controlled, open-label study and the second phase conducted after the first year for another 52 weeks was an extended, open-label, observational study. The study was conducted at 11 clinical sites in China. Here, we report the first phase study results from baseline up to week 52.

In the first phase, all subjects were randomized 3:1 to daily subcutaneous injections of rhGH 0.05 mg/kg/day (Jintropin^®^, GeneScience Pharmaceuticals, Changchun, China) or no treatment for 52 weeks or until unacceptable toxicity or investigator decision. There was no positive control group in this study because GH was not approved for ISS in China at the start of this study. Block randomization method was performed using SAS version 9.4 (SAS Institute, Cary, NC, USA). All subjects eligible for the study were given a random number in the order of enrollment, and a central randomization system was used to determine whether the subject was allocated to the treatment or control group. The random unique identifier generated for each subject was used throughout the study.

According to the U.S. FDA, the maximum dose of GH approved for the treatment of ISS in children is 0.47 mg/kg/week (equivalent to 0.067 mg/kg/day or 0.2 IU/kg/day). The Chinese Society of Pediatric Endocrinology and Metabolism (CSPEM) recommends children with ISS should receive rhGH at a dose of 0.043–0.07 mg/kg/day, equivalent to 0.125–0.2 IU/kg/day (9). In this study, children in the treatment group were given somatropin 0.05 mg/kg/day subcutaneously, which was equivalent to 0.15 IU/kg/day, lower than the U.S. approved dose and within the CSPEM recommended dose.

The study was carried out according to the Declaration of Helsinki and complied with the standards of Good Clinical Practice. Written informed consent from subjects, parents, or guardians was obtained prior to enrollment. The protocol was reviewed and approved by the Ethics Committee of each investigation site.

### 2.3 Outcomes and Assessment

All subjects underwent a total of 6 visits to the clinic throughout the first phase of the study at baseline and weeks 4, 13, 26, 39, and 52. The primary objective of this study was to compare the treatment improvement in HT-SDS with control at week 52. The secondary objective was to determine the improvement in annual HV at week 52 with treatment.

The primary outcome measure was △HT-SDS for chronological age (CA) from baseline at week 52. Other secondary outcome measures included △HT-SDS for CA at weeks 4, 13, 26, and 39; △height from baseline; △BA/CA; △HV; and △IGF-1 SDS. Safety was monitored throughout the study and assessed based on reported AEs, physical examinations, vital signs, laboratory test results (e.g., blood, urine, antidrug antibodies, thyroid function, fasting blood glucose), whole-spine X-rays, and electrocardiograms (ECGs).

GH stimulation tests and pituitary MRIs were performed within 1 year before randomization at the investigation site where the subjects were screened. All other tests were performed within 8 weeks before randomization at the participating site. Predicted adult height (PAH) was also assessed using the China05 method (The Standards of Skeletal Maturity of Hand and Wrist for Chinese–China 05 and its application) (10). BA radiography was performed using the TW3-AI method (11) and the results were collated and analyzed by a qualified researcher appointed by the principal investigator at the Children’s Hospital of Zhejiang University School of Medicine. IGF-1 and IGF-binding protein 3 (IGFBP-3) serum were analyzed at a central laboratory. Subjects in the treatment group were screened for antidrug and neutralizing antibodies at baseline and weeks 26 and 52.

### 2.4 Statistical Analysis

All statistical analyses in the first phase of the study were performed using SAS version 9.4.

This study was designed to demonstrate the superiority of somatropin versus no treatment in terms of improving HT-SDS. Based on previous research and the investigators’ decision, the predetermined difference in mean change of HT-SDS in the experimental and control groups after 52 weeks was set at δ = 0.5. Assuming a combined variance of 1.44, type I error α = 0.025, type II error β = 0.15, and a power of 0.85, the required sample sizes for the treatment and control groups were not to be less than 210 and 70 subjects, respectively. To ensure that the results were statistically robust, conformed to the minimum number of patients for a phase 3 study required by China’s National Medical Products Administration, and accounted for a 20% dropout rate, a total of 480 subjects (somatropin: 360; untreated control: 120) were recruited.

The full analysis set (FAS) of the first study phase was defined as all subjects who received at least 1 dose of study drug, had baseline assessments, and had at least 1 postbaseline assessment evaluated after randomization, according to the intention-to-treat (ITT) principle. All missing data were imputed using the last-observation-carried-forward method. The per-protocol set (PPS) was a subset of the FAS that included all subjects without any major protocol deviations. The FAS was the main data set for the evaluation of efficacy. Both ITT and PPS were analyzed to prevent selection bias. Safety data analyses were performed on a safety set (SS) that included all subjects who had received the study drug at least once in the treatment group and all subjects in the control group after randomization.

Data were presented as mean ± SD for quantitative and efficacy variables, and frequency and percentage for qualitative variables. Descriptive statistics were used to summarize baseline characteristics. *P* < 0.05 was considered statistically significant. Within-group comparisons were assessed using the paired t test and Wilcoxon rank-sum test. Intergroup comparisons were performed using analysis of covariance (ANCOVA). The change from baseline efficacy endpoints at week 52 was tested for superiority of somatropin to no treatment using least squares mean (LSM) difference. There is evidence of superiority if the 95% confidence interval (CI) for the treatment effect lies entirely above zero. AEs were summarized descriptively by severity and relationship to somatropin.

A sensitivity analysis was performed in the first phase with baseline HT-SDS and study group as the fixed effects, and the center was used as the random effect. Comparison between groups was performed using the mixed-effects model. The factors associated with ΔHT-SDS, ΔHV, and ΔPAH in response to GH treatment were determined in separate multivariate linear regression analyses.

## 3 Results

### 3.1 Subject Baseline Characteristics and Demographics

**Figure 1:**
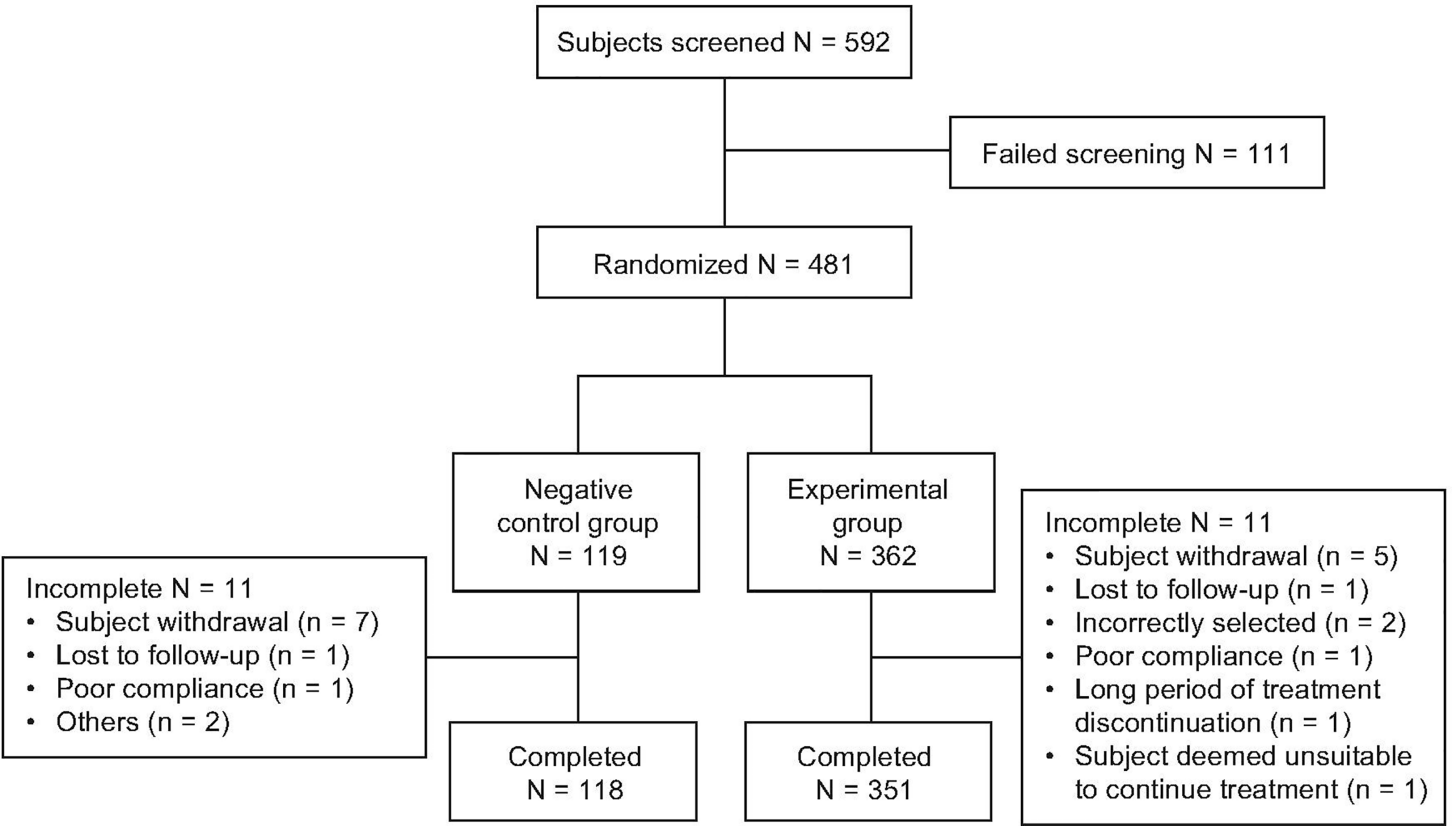
Patient flow throughout the trial.

A total of 592 subjects were screened, of whom 481 were randomized 3:1 to somatropin (n = 362) and untreated control (n = 119) (**Figure 1**). Three hundred and fifty-one (97.0%) and 108 (90.8%) subjects in the treatment and control groups, respectively, competed the study. Twenty-two subjects (11 from each group) dropped out early, the most common reason being “withdrawal of consent” (n = 12). The numbers of subjects included in the FAS, PPS, and SS were 472 (98.1%), 459 (95.4%), and 481 (100%), respectively.

The demographic information and baseline characteristics of the study subjects are presented in **Table 1**. The mean CA of the subjects was 5.8 ± 1.55 years (range: 4.0–9.0 years). The mean height was 106.0 ± 8.14 cm, the mean weight was 17.0 ± 2.93 kg, and the mean body mass index was 15.1 ± 1.26. Baseline HT-SDS was similar in both the treatment and control groups (somatropin: –2.64 ± 0.41; untreated control: –2.67 ± 0.44). Pretreatment HV did not differ between the groups. The percentage of subjects who were compliant with treatment was 98.03% ± 2.45%.

**Table 1:** Patient demographics and baseline characteristics of the FAS.

	Untreated control (n = 119)	Somatropin 0.05 mg/kg/day (n = 362)	Total (N = 481)
Chronological age, year	6.0 ± 1.67	5.8 ± 1.51	5.8 ± 1.55
Gender			
Male, n (%)	63 (56.3)	224 (62.2)	287 (60.8)
Female, n (%)	49 (43.8)	136 (37.8)	185 (39.2)
Height, cm	106.81 ± 8.81	105.75 ± 7.91	106.00 ± 8.14
Weight, kg	17.30 ± 3.02	16.90 ± 2.90	17.00 ± 2.93
BMI, kg/m^2^	15.10 ± 1.27	15.10 ± 1.27	15.10 ± 1.26
Ethnicity			
Han (%)	108 (96.4)	350 (97.2)	458 (97.0)
Others (%)	4 (3.6)	10 (2.8)	14 (3.0)
HT-SDS	–2.67 ± 0.44	–2.64 ± 0.41	–2.65 ± 0.42
BA/CA	0.81 ± 0.15	0.81 ± 0.15	0.81 ± 0.15
HV, cm/year	5.46 ± 5.10	5.00 ± 3.38	5.06 ± 3.85
IGF-1 SDS	–0.72 ± 0.95	–0.49 ± 0.99	–

BA, bone age; BMI, body mass index; CA, chronological age; FAS, full analysis set; HT-SDS, height standard deviation score; HV, height velocity; IGF-1 SDS, insulin-like growth factor-1 standard deviation score.

### 3.2 Primary and Secondary Endpoints

#### 3.2.1 HT-SDS and △HT-SDS

**Figure 2:**
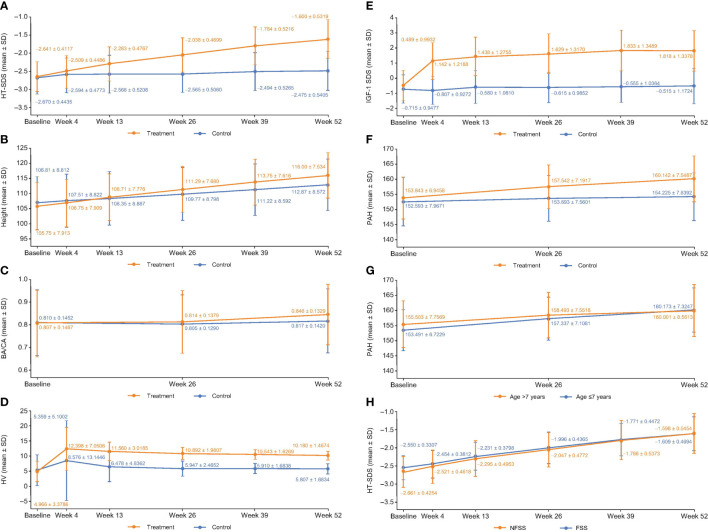
**(A)** HT-SDS, **(B)** height, **(C)** BA/CA, **(D)** HV, **(E)** ΔIGF-1 SDS, **(F)** PAH, **(G)** PAH stratified by age and, **(H)** HT-SDS for FSS and NFSS of the FAS at each evaluable time point. Δ, change in; BA, bone age; CA, chronological age; FAS, full analysis set; FSS, familial short stature; HT-SDS, height standard deviation score; HV, height velocity; IGFBP-3, insulin-like growth factor binding protein 3; IGF-1 SDS, insulin-like growth factor-1 standard deviation score; NFSS, nonfamilial short stature; PAH, predicted adult height; SD, standard deviation.

At week 52, the HT-SDSs in the treatment and control groups were –1.60 ± 0.53 and –2.48 ± 0.54, respectively (**Figure 2A**). The mean △HT-SDS at week 52 relative to baseline was 1.04 ± 0.31 in the treatment group and 0.20 ± 0.33 in the control group, showing a statistically significant difference between the 2 groups (*P* < 0.001, **Table 2**). △HT-SDS at all evaluable time points from baseline was statistically significant for both study groups (*P* < 0.001). The LSM difference in △HT-SDS at week 52 between the treatment and control groups was 0.85 (95% CI 0.78–0.91), indicating superiority of treatment over control. Subjects in the treatment group converged toward the normal range (HT-SDS ≥ –2) at the end of 52 weeks. Greater ΔHT-SDS at week 52 was observed in children aged ≤7 years than those who were aged >7 years (1.08 ± 0.31 vs. 0.85 ± 0.24).

**Table 2:** Efficacy outcome measures of the FAS.

	Untreated control (n = 112)	Somatropin 0.05 mg/kg/day (n = 360)
ΔHT-SDS		
Week 4	0.08 ± 0.22	0.13 ± 0.14
Week 13	0.10 ± 0.28	0.36 ± 0.21
Week26	0.11 ± 0.25	0.60 ± 0.22
Week39	0.18 ± 0.30	0.86 ± 0.28
Week52	0.20 ± 0.33	1.04 ± 0.31
ΔHeight, cm		
Week4	0.70 ± 1.05	1.00 ± 0.58
Week13	1.67 ± 1.22	2.93 ± 0.80
Week26	3.04 ± 1.24	5.51 ± 1.02
Week39	4.51 ± 1.35	7.98 ± 1.25
Week52	5.85 ± 1.80	10.19 ± 1.47
ΔPAH, cm		
Week26	1.13 ± 4.09	3.66 ± 4.08
Week52	1.55 ± 4.96	6.28 ± 4.84
ΔBA/CA		
Week26	–0.007 ± 0.070	0.009 ± 0.070
Week52	0.004 ± 0.100	0.040 ± 0.090
ΔHV, cm/year		
Week4	3.12 ± 14.83	7.43 ± 8.39
Week13	1.05 ± 7.57	6.58 ± 4.92
Week26	0.55 ± 5.97	5.91 ± 4.10
Week39	0.51 ± 5.61	5.56 ± 3.81
Week52	0.75 ± 4.34	5.17 ± 3.70
ΔIGF-1 SDS		
Week4	–0.10 ± 0.71	1.62 ± 0.92
Week13	0.15 ± 1.02	1.92 ± 1.06
Week26	0.10 ± 0.79	2.12 ± 1.11
Week39	0.15 ± 0.87	2.33 ± 1.15
Week52	0.22 ± 0.98	2.31 ± 1.20

Δ, change in; BA, bone age; CA, chronological age; FAS, full analysis set; HT-SDS, height standard deviation score; HV, height velocity; IGF-1 SDS, insulin-like growth factor-1 standard deviation score; PAH, predicted adult height.

Efficacy in the PPS also showed similar results. HT-SDS was –1.58 ± 0.51 and –2.64 ± 0.41 in the treatment and control groups, respectively. Mean △HT-SDS was 1.06 ± 0.30 with treatment and 0.18 ± 0.27 in the control group, with the difference between both groups statistically significant (*P* < 0.001). The LSM difference in △HT-SDS between treatment and control was 0.88 (95% CI 0.82–0.94).

#### 3.2.2 Height

Increase in mean height was observed in both study groups across all evaluable time points (**Figure 2B**), with those in the treatment group experiencing larger gain in height compared with untreated subjects. △height was statistically significantly higher in the treatment group compared with control at all time points (*P* < 0.001). At week 52, mean △height from baseline in the treatment group was 10.19 ± 1.47 cm and 5.85 ± 1.80 cm in the control group (**Table 2**). The LSM difference in the △height between the treatment and control groups was 4.27 (95% CI 3.95–4.59).

#### 3.2.3 BA/CA

At week 52, the BA/CA ratios were 0.85 ± 0.13 and 0.82 ± 0.14 in the treatment and control groups, respectively (**Figure 2C**). The mean △BA/CA ratio significantly increased from baseline at week 52 with treatment (0.04 ± 0.09; *P* < 0.001, **Table 2**). There was a significant difference between groups in △BA/CA at week 52 (*P* < 0.001); the LSM difference between the treatment and control groups at weeks 26 and 52 was 0.014 (95% CI –0.0003 to 0.0287) and 0.035 (95% CI 0.017–0.052), respectively. At week 52, 95% CI was more than 0, indicating that treatment had an effect on bone maturation.

#### 3.2.4 HV

Mean HV increased sharply in the first month of treatment from 4.97 ± 3.38 cm/year at baseline to 12.40 ± 7.05 cm/year at week 4, before plateauing (**Figure 2D**). Interestingly, a similar trend was also observed in the control group (**Figure 2D**). The annualized HVs at week 52 in the treatment and control groups were 10.18 ± 1.47 cm/year and 5.81 ± 1.68 cm/year, respectively (**Figure 2D**). △HVs at 52 weeks were 5.17 ± 3.70 cm/year and 0.75 ± 4.34 cm/year in the treatment and control groups, respectively (**Table 2**). Compared with baseline, all study groups were associated with a significant increase in HV at all time points. The LSM of △HV at week 52 between the treatment and control groups was 4.42 cm/year (95% CI 4.10–4.75), demonstrating superiority in terms of increment in HV with treatment.

There was not much difference in HV between children aged ≤7 years and those aged >7 years (5.17 ± 3.89 cm/year vs. 5.16 ± 2.63 cm/year). However, the LSM difference between >7-year-olds and ≤7-year-olds in terms of ΔHV by ANCOVA was –1.12 cm/year (95% CI –1.48 to –0.75).

#### 3.2.5 IGF-1 SDS

IGF-1 SDS increased sharply from –0.49 ± 0.99 at baseline to 1.14 ± 1.22 at week 4 with treatment and progressed steadily before plateauing at week 39 (**Figure 2E**). At week 52, statistically significant △IGF-1 SDS from baseline was observed in the treatment (2.31 ± 1.20; *P* < 0.001) and control groups (0.22 ± 0.98; *P* = 0.021) (**Table 2**). Treatment differed significantly compared with control at all time points (*P* < 0.001); the LSM difference between treatment and control was 2.16 (95% CI 1.92–2.41) at week 52. The 95% CI of LSM difference was more than 0 from week 4 through week 52, indicating superiority in the treatment group throughout the study.

### 3.3 Additional Assessment

#### 3.3.1 PAH

PAH was analyzed since the study did not follow up with the subjects until adult height was achieved. The mean PAH at the start of treatment in the treatment group was 153.84 ± 6.95 cm and reached a mean of 160.14 ± 7.55 cm at the end of treatment (*P* < 0.001; **Figure 2F**). In contrast, the PAH for subjects in the control group was 152.59 ± 7.96 cm at baseline and 154.22 ± 7.84 cm at 52 weeks (*P* = 0.002; **Figure 2F**). ΔPAHs in the treatment and control groups were 6.28 ± 4.81 cm and 1.55 ± 4.96 cm, respectively (**Table 2**). The LSM in ΔPAHs at week 52 between the treatment and control groups was 4.93 (95% CI 3.91–5.96).

PAH was further stratified by age to assess if age of initiation had an impact on the efficacy of GH treatment. ΔPAH at week 52 was 6.67 ± 4.96 cm in children aged ≤7 years and 4.50 ± 3.81 cm in children aged >7 years, suggesting that a greater gain in height was observed in children aged ≤7 years (**Figure 2G**). The LSM difference in ΔPAH between the treatment and control groups in children aged ≤7 years was 5.51 (95% CI 4.32–6.70) and 2.77 (95% CI 0.90–4.65) in children aged >7 years.

#### 3.3.2 Familial Short Stature

ISS is a heterogenous condition covering children with familial short stature (FSS) and nonfamilial short stature (NFSS). FSS is defined as a child with short stature compared with the relevant population, but remains within the expected target height range for the family, with 1 parent HT-SDS < –2 (1). Here, subgroups of FSS and NFSS were analyzed to determine the impact of GH treatment. At 52 weeks, in GH-treated subjects, ΔHT-SDS from baseline was 0.94 ± 0.30 in the FSS group and 1.06 ± 0.31 in the NFSS group (both *P* < 0.001). In the FSS group, HT-SDS was –1.61 ± 0.47 and –2.40 ± 0.45 in the treatment and control groups, respectively, whereas in the NFSS group, HT-SDS was –1.60 ± 0.55 and –2.49 ± 0.56 in the treatment and control groups, respectively, at week 52. **Figure 2H** compares the HT-SDS between the FSS and NFSS groups. There was significant improvement in terms of HT-SDS (*P* < 0.001) in the NFSS group compared with the FSS group at the end of 52 weeks (**Figure 2H**). The LSM difference in ΔHT SDS between FSS and NFSS was –0.11 (95% CI –0.19 to –0.04). PAH was 4.99 ± 4.88 and 0.14 ± 4.03 in the FSS treatment and control groups, respectively, whereas in the NFSS group, PAH was 6.56 ± 4.80 and 1.83 ± 5.10 in the treatment and control groups, respectively, at week 52. The LSM difference in ΔPAH at week 52 between FSS and NFSS was –1.36 (95% CI –2.49 to –0.23). Similar trends were also observed with HV at 52 weeks, with the FSS group and the NFSS group achieving mean HV of 9.75 ± 1.46 cm/year and 10.27 ± 1.45 cm/year with treatment, respectively. The LSM difference between FSS and NFSS in ΔHV at week 52 was –0.48 (95% CI –0.86 to –0.10).

### 3.4 Safety

The frequencies of total treatment-emergent adverse events (TEAEs) were similar in both study groups (treatment vs. control: 89.8% vs. 82.4%, **Table 3**). Most TEAEs were mild to moderate in severity. The most common TEAEs in the treatment group were upper respiratory tract infection (66.0%), fever (19.6%), cough (10.8%), bronchitis (5.5%), respiratory tract infection (7.2%), rhinitis (2.8%), and indigestion (2.5%). One subject withdrew from the study due to neutropenia, which was deemed unrelated to treatment.

**Table 3:** Adverse events of the SS.

	Untreated control (n = 119) n (%)	Somatropin 0.05 mg/kg/day (n = 362) n (%)	Total (N = 481) n (%)
Total TEAEs	98 (82.4)	325 (89.8)	426 (88.6)
Total TRAEs	0 (0.0)	23 (6.4)	23 (4.8)
SAEs	6 (5.0)	19 (5.2)	25 (5.2)
Treatment suspension due to TEAEs	0 (0.0)	203 (56.1)	203 (42.2)
Treatment suspension due to TRAEs	0 (0.0)	2 (0.6)	2 (0.4)
Treatment suspension due to SAEs	0 (0.0)	13 (3.6)	13 (2.7)
TEAEs occurring in ≥5% of subjects in any group			
Upper respiratory tract infection	78 (65.5)	239 (66.0)	317 (65.9)
Fever	10 (8.4)	71 (19.6)	81 (16.8)
Cough	10 (8.4)	39 (10.8)	49 (10.2)
Bronchitis	12 (10.1)	20 (5.5)	32 (6.7)
Respiratory tract infection	5 (4.2)	26 (7.2)	31 (6.4)
Rhinitis	8 (6.7)	10 (2.8)	18 (3.7)
Indigestion	7 (5.9)	9 (2.5)	16 (3.3)

SAEs, serious adverse events; SS, safety set; TEAEs, treatment-emergent adverse events; TRAEs, treatment-related adverse events.

AEs reported in 23 (6.4%) subjects in the treatment group were considered related to treatment. They were all mild to moderate in severity. Of note, 4 subjects experienced elevated thyroid-stimulating hormone, 3 had scoliosis, elevated blood glucose level and rash occurred in 2 subjects each, and hypersensitivity and hypothyroidism were reported in 1 subject each. All but 3 subjects (1 case of hypothyroidism, scoliosis, and extremity pain each) recovered from the drug-related TEAEs. Serious AEs (SAEs) occurred in 25 subjects (treatment: 19 [5.2%], control: 6 [5.0%]) and were deemed unrelated to treatment. All subjects recovered from the SAEs. No deaths were reported with GH treatment in the study.

The numbers of subjects with IGF-1 SDS more than +2 in the treatment and control groups at week 52 were 154 (42.5%) and 2 (1.7%), respectively. IGF-1/IGFBP-3 ratios in the treatment and control groups were 0.19 ± 0.05 and 0.13 ± 0.04, respectively. Abnormal ECGs were reported in 7 (1.9%) subjects in the treatment group and 2 (1.6%) subjects in the control group. Whole-spine X-ray examination was deemed abnormal in 13 subjects in the treatment group and 4 subjects in the control group. There were generally no unexpected safety issues with respect to clinical laboratory examinations, vital signs, and physical examinations.

The numbers of subjects detected with antidrug antibodies at baseline, week 26, and 52 were 0 (0.0%), 5 (1.4%), and 10 (2.8%), respectively; they all tested negative for neutralizing antibodies.

## 4 Discussion

Several studies have been conducted to investigate the benefits of rhGH therapy in children with ISS; however, only a few were randomized with a negative control or placebo to compare the effect of treatment on height outcomes (12–15). The purpose of this study was to evaluate the safety and efficacy of daily somatropin at a dose of 0.05 mg/kg/day in Chinese children with ISS, and to assess the superiority of treatment over control. Overall, the results for the PPS were consistent with the FAS across all outcome measures, confirming the robustness of these data. The parallel study design and large number of children recruited made this study one of the few that provided objective evidence of the effects of daily rhGH treatment in the Asian population.

Greater improvement in HT-SDS was observed in the treatment group than in the control group at week 52 (–1.60 ± 0.53 vs. –2.48 ± 0.54). The mean HT-SDS of subjects who received somatropin reached normal range (≥ –2.25) after a year of treatment, indicating that GH treatment has a positive impact on growth. These subjects achieved a mean height of 3.13 cm taller than untreated subjects. In a meta-analysis of 6 randomized and 4 nonrandomized controlled trials evaluating the effect of short-term GH therapy in children with ISS, the difference in HT-SDS between the treatment and control group was reported to be 0.60 after a year (16). HV was significantly greater in the GH-treated group than in controls after 1 year of treatment, with the pooled estimate for the difference between both groups being 2.86 ± 0.37 cm/year (16). Of note, the age ranges of the analyzed children were older, with a few studies including pubertal children. This meta-analysis provided evidence that 1 year of GH therapy can increase HV and HT-SDS.

The results of our study were consistent with more recent studies in the Asian population, showing improvements in auxological variables in terms of HT-SDS and HV. In a phase 3, randomized, controlled trial, Chung et al. reported an increase in 6-month HV from 5.63 ± 1.62 cm/year at baseline to 10.08 ± 1.92 cm/year with Saizen^®^ 0.067 mg/kg/day. The difference in △HV between treatment and control was 3.47 cm/year (95% CI 2.51–5.00; *P* < 0.0001). △HT-SDS was 0.96 at 12 months with treatment (12). Although the subjects recruited were slightly older and the treatment dose was higher, their results were comparable with ours. Similar benefits were observed in another phase 3 study conducted by Kim et al. (14). In this study, HV and HT-SDS were 10.68 ± 1.95 cm/year and 0.63 ± 0.16, respectively, after 6 months of Growtropin^®^-II 0.37 mg/kg/week, which was equivalent to 0.05 mg/kg/day (14). In an open-label study, Eutropin^®^ 0.37 mg/kg/week for 26 weeks was able to achieve a HV of 6.36 ± 3.36 cm/year and the gain in HT-SDS was 0.57 ± 0.27 (17). The short-term benefit of GH treatment was also demonstrated in a real-world observational study of 2,596 subjects with ISS with a mean age of 11.5 years and a baseline HT-SDS of –2.3 ± 0.8 (18). 1-year of GH treatment improved HT-SDS by 0.61 ± 0.33 (18). These studies demonstrated that short-term GH treatment was able to achieve growth enhancement in prepubertal children with ISS.

Daily administration of rhGH in prepubertal children with ISS during the 52-week treatment period significantly increased ΔPAH, HV, and IGF-1 SDS compared with control at all time points. Of note, the improved PAH could be taken to denote that short-term treatment had a beneficial effect on final height. While BA/CA increased with GH treatment compared with control, it remained <1 after 52 weeks of treatment. Given the large sample size, it was easier to achieve statistical significance with bone maturation as it increased from 0.81 ± 0.15 at baseline to 0.85 ± 0.13 at week 52. △BA/CA was 0.04 ± 0.09, indicating slight progression of BA. The LSM difference also showed that GH treatment increased △BA/CA compared with control. However, 1 study from Korea reported no significant difference in ΔBA from baseline with treatment compared with control (14). The effect of treatment on BA/CA varies, 1 study reported a ratio of 1.06 ± 1.00 after 3 years of treatment, while another 0.93 ± 0.11 with 1 year of treatment (19, 20). The inclusion of children during peripuberty or puberty may influence BA progression and BA/CA ratio because of the effects of sex steroids. In our study, all enrolled children were prepubertal at baseline, and only 19 (4.0%; 13 [3.6%] in the treatment group and 6 [5.4%] in the control group) advanced to a higher Tanner stage by week 52, which was unlikely to confound the efficacy assessments. Nonetheless, a delay in BA at the onset of treatment was associated with greater BA progression in the first year of therapy and longer-term treatment may gradually increase BA/CA ratio to 1, enabling BA to catch up with CA (21, 22).

Subjects were further stratified by age. Children aged 7 years or younger showed larger increments in ΔHT-SDS, △HV, and ΔPAH compared with those who were older than 7 years, suggesting that starting GH treatment earlier may yield better growth outcomes. Further study is warranted to elucidate the optimal age of treatment. In a retrospective cohort study by Ranke et al., height achieved and gain in HT-SDS depended on the age at which GH was initiated, supporting the notion that starting GH treatment earlier yielded a better response (23).

It has been suggested that ISS can be subdivided into FSS and NFSS. FSS refers to children with a normal growth velocity and growing in a normal trajectory toward their midparental height range but are short compared to the reference population (24). In our subgroup analysis, treated subjects categorized as NFSS had a higher HT-SDS, PAH, and HV compared with those in the FSS group. Similarly, Sotos and Tokar conducted a retrospective analysis to compare FSS and NFSS, and reported a favorable height gain in the latter (25). Similarly, earlier studies by Wit et al. and Albertsson-Wikland et al. also showed that children with NFSS responded better to GH treatment than those with FSS (26, 27), as FSS is a condition believed to be caused by small contributions of multiple genes. The smaller benefit observed in children with FSS compared with NFSS may be attributed to lower GH sensitivity, GH resistance, or mutations in the IGF-1 gene (24). Based on this sub-analysis, it may be useful to categorize subjects with ISS into FSS and NFSS so as to better predict their growth outcomes with GH treatment. Further research is warranted to validate this observation.

Daily somatropin 0.05 mg/kg/day had a favorable safety profile throughout the 52-week study period, apart from the subject who experienced elevated alanine aminotransferase. The subject was treated with cough syrup prior to the GH treatment and there were no other abnormal signs or symptoms. It was not clear whether the increase in alanine aminotransferase was due to an infection or the cough syrup, nevertheless, the subject recovered without needing further intervention. In 1 study, Quigley et al. also observed AEs such as scoliosis, hypothyroidism, and changes in carbohydrate metabolism with GH treatment in pediatric patients with ISS based on a dosing range of 0.22–0.37 mg/kg/week (28). Interestingly, otitis media, which was not present in our study, was reported in 8% of patients (28). Leschek et al. reported scoliosis in 7 patients who received GH treatment at a dose of 0.22 mg/kg/wk, 3 more than placebo (15). Another study reported mild pruritus on the injection site, which was absent in this present study, as the only adverse event related to treatment spontaneously resolved without any intervention (17). The incidences of TEAEs reported in both study groups were similar. All SAEs that occurred during the study, such as upper respiratory tract infection, bronchitis, and tonsilitis, were deemed unrelated to the treatment. Elevated IGF-1 levels have been associated with the development of cancer (29). While the mean IGF-1 SDS in the treatment group of our study was 1.82 ± 1.34 at week 52, 42.5% of subjects had IGF-SDS levels of more than +2, which was above the normal range using age-appropriate reference standards. Although there has been no evidence so far to suggest increased risk of some cancers in later life with the use of GH, it is appropriate to monitor IGF-1 levels and adjust and tailor the dose where necessary, especially for those who are receiving long-term treatment. Antidrug antibodies to somatropin that were detected in some children had no effect on the efficacy and safety of treatment, as their data showed consistent results with those of antibody-negative subjects.

The strength of this study is the inclusion of a negative control group, giving confidence that any efficacy and safety effects may be attributable to daily somatropin at 0.05 mg/kg/day. While randomized, controlled trials provide the most robust evidence, they may not reflect what occurs in the real world. Hence, the ongoing, open-extension, observational study will shed light on whether the clinical benefit of somatropin is sustained following another year of individualized treatment.

There are several limitations of this study. It does not present a full picture of short-term treatment on adult height. It also does not elucidate the long-term efficacy of somatropin. As such, longer treatment and follow-up are warranted. This is supported by a recent observational study in China where a longer GH-treatment course of ≥2 years yielded better efficacy compared with shorter treatment courses in terms of △HT-SDS in children with ISS, despite administering during peripuberty (≥2 years vs. 1–2 years vs. 6–12 months vs. 3–6 months: 1.54 ± 1.23 vs. 1.01 ± 1.31 vs. 1.00 ± 1.27 vs. 1.30 ± 1.09) (6). The extended, open-label, observation study of this phase 3 trial will provide a better understanding on the longer-term effects of somatropin in children with ISS in China.

In conclusion, daily somatropin at a dose of 0.05 mg/kg/day demonstrated superiority to no treatment in terms of gain in HT-SDS and HV increment. There was a significant increase in height gain, PAH, and IGF-SDS after 52 weeks of treatment in prepubertal children with ISS. somatropin was well tolerated with a favorable safety profile.

## Data Availability Statement

The original contributions presented in the study are included in the article/supplementary material. Further inquiries can be directed to the corresponding author.

## Ethics Statement

The studies involving human participants were reviewed and approved by The Children’s Hospital of Zhejiang University School of Medicine (No.2018-IEC-003). Written informed consent to participate in this study was provided by the participants’ legal guardian/next of kin.

## Author Contributions

JF had full access to all of the data in the study and takes responsibility for the integrity of the data and the accuracy of the data analysis. JF and JY conceptualized and designed the clinical study, compiled and analyzed the data. HW, GZ, YX, HD, WG, YL, LC, FL, YZ and HG carried out the clinical assessments. JY wrote the manuscript with critical input from the other authors. All authors approved the final version of the manuscript.

## Funding

This research was partly funded by GeneScience Pharmaceuticals Co., Ltd. The funder did not participate in the writing of the manuscript or the collection, analysis, and interpretation of the data.

## Conflict of Interest

The authors declare that the research was conducted in the absence of any commercial or financial relationships that could be construed as a potential conflict of interest.

## Publisher’s Note

All claims expressed in this article are solely those of the authors and do not necessarily represent those of their affiliated organizations, or those of the publisher, the editors and the reviewers. Any product that may be evaluated in this article, or claim that may be made by its manufacturer, is not guaranteed or endorsed by the publisher.

## Abbreviations

△, change in; AE, adverse event; ANCOVA, analysis of covariance; BA, bone age; CA, chronological age; CI, confidence interval; CSPEM, Chinese Society of Pediatric Endocrinology and Metabolism; ECG, electrocardiogram; FAS, full analysis set; FSS, familial short stature; GH, growth hormone; GH-IGF-1, growth hormone-insulin-like growth factor 1; IGFBP-3, IGF-binding protein 3; ITT, intention-to-treat; HT-SDS, height standard deviation score; HV, height velocity; ISS, idiopathic short stature; LSM, least squares mean; MRI, magnetic resonance imaging; NFSS, nonfamilial short stature; PAH, predicted adult height; PPS per-protocol set; rhGH, recombinant human GH; SAE, serious adverse event; SD, standard deviation; SDS, standard deviation score; SHOX, short stature homeobox-containing; SS, safety set; TEAE, treatment-emergent adverse event.
